# Crystal structure of *Thermus thermophilus* methylenetetrahydrofolate dehydrogenase and determinants of thermostability

**DOI:** 10.1371/journal.pone.0232959

**Published:** 2020-05-13

**Authors:** Fernando Maiello, Gloria Gallo, Camila Coelho, Fernanda Sucharski, Leon Hardy, Martin Würtele

**Affiliations:** 1 Department of Science and Technology, Federal University of São Paulo, São José dos Campos, Brazil; 2 Department of Physics, University of South Florida, Tampa, FL, United States of America; CIC bioGUNE, SPAIN

## Abstract

The elucidation of mechanisms behind the thermostability of proteins is extremely important both from the theoretical and applied perspective. Here we report the crystal structure of methylenetetrahydrofolate dehydrogenase (MTHFD) from *Thermus thermophilus* HB8, a thermophilic model organism. Molecular dynamics trajectory analysis of this protein at different temperatures (303 K, 333 K and 363 K) was compared with homologous proteins from the less temperature resistant organism *Thermoplasma acidophilum* and the mesophilic organism *Acinetobacter baumannii* using several data reduction techniques like principal component analysis (PCA), residue interaction network (RIN) analysis and rotamer analysis. These methods enabled the determination of important residues for the thermostability of this enzyme. The description of rotamer distributions by Gini coefficients and Kullback–Leibler (KL) divergence both revealed significant correlations with temperature. The emerging view seems to indicate that a static salt bridge/charged residue network plays a fundamental role in the temperature resistance of *Thermus thermophilus* MTHFD by enhancing both electrostatic interactions and entropic energy dispersion. Furthermore, this analysis uncovered a relationship between residue mutations and evolutionary pressure acting on thermophilic organisms and thus could be of use for the design of future thermostable enzymes.

## Introduction

While considerable experimental and theoretical advances have been made, the precise underlying mechanisms of protein thermostability remain elusive. However, several important features of thermophilic protein stabilization mechanisms have been identified. Thermostability of proteins appears to involve enhancing the number of salt bridges, hydrogen bonds and charged amino acids like arginines [1–3] and a better hydrophobic packing and/or larger hydrophobic core including more amino acids like tyrosines [4–6]. Certainly, thermostability can be thus linked to a multitude of different mechanisms with no single mechanism uniquely determining the thermostability of all proteins. One emerging motif is the possible presence of a network of stabilizing charged residues in thermophilic and especially extreme thermophilic proteins [7,8].

Recent studies have analyzed thermostability using sampling simulation techniques like molecular dynamics (MD) [9,10]. Complemented with known analysis tools, these techniques have been fruitful in characterizing the dynamic rather than just the static causes of thermostability. MD analysis has been complemented by more global analyses of trajectories, including normal-mode analysis (NMA) [11], principal component analysis (PCA) [12] and residue interaction network (RIN) analysis [13,14].

**Fig 1:**
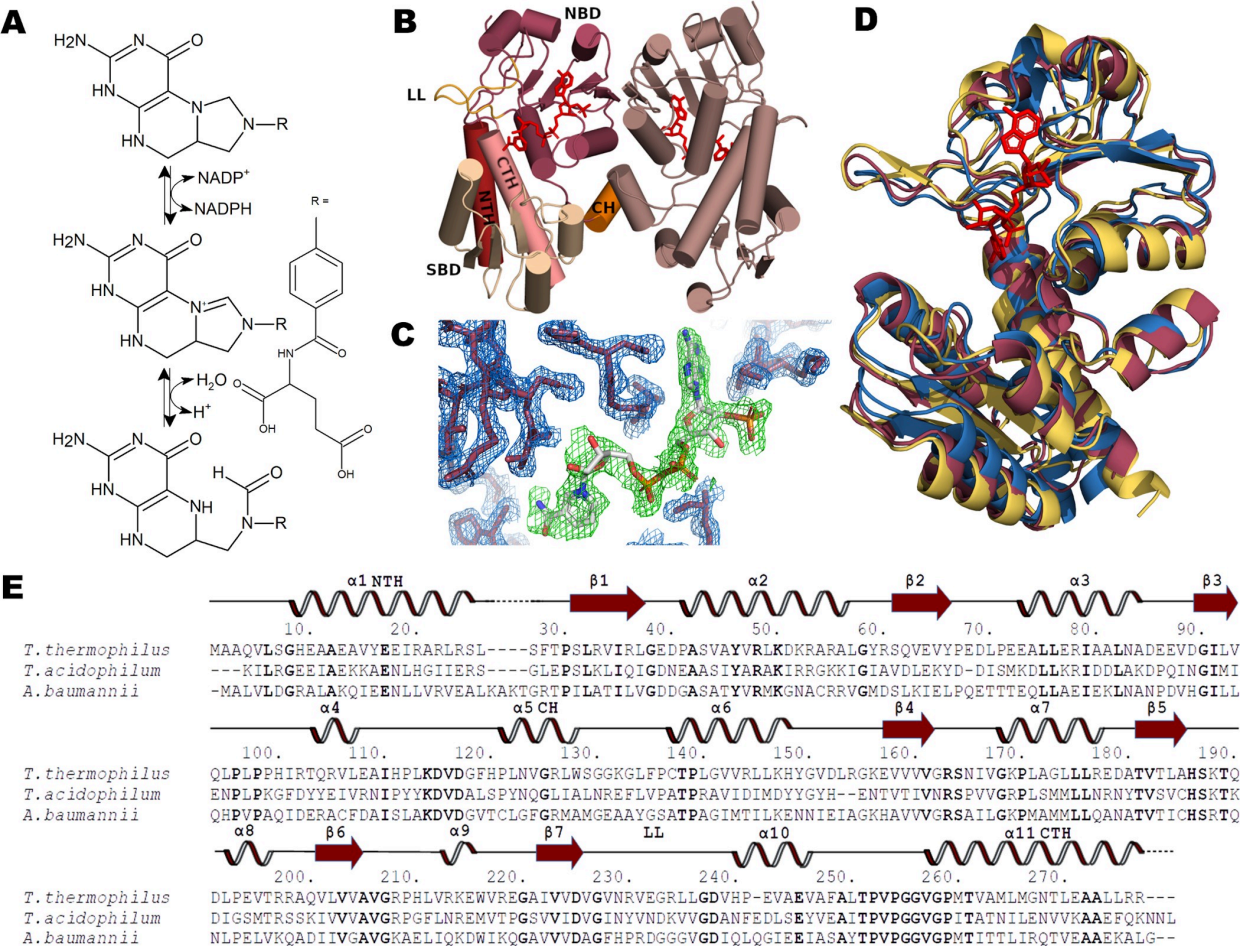
MTHFD structure and function. (A) MTHFD catalyzes the conversion of 5,10‐methylenetetrahydrofolate via 5,10-methenyltetrahydrofolate to 10-formyltetrahydrofolate in a NADP^+^ dependent manner. (B) Secondary structural elements of the *T*. *thermophilus* MTHFD dimer. Important structural elements are indicated. LL: lid loop, NTH: N-terminal helix, SBD: substrate binding domain, CH: connector helix, NBD: nucleotide binding domain, CTH: C-terminal helix. (C) 2F_o_-F_c_ electron density map at the 1.0 σ level of the active site of *T*. *thermophilus* MTHFD showing the NADP^+^ nucleotide. (D) Alignment of *Thermus thermophilus* (red) *Thermoplasma acidophilum* (yellow) *Acinetobacter baumannii* (blue) crystal structures. (E) Amino acid sequence alignment of MTHFD from *T*. *thermophilus*, *T*. *acidophilum* and *A*. *baumannii* with secondary structure elements (α-helices, β-strands as arrows) of *T*. *thermophilus* indicated above sequences. Numbering (indicated by dots) refers to the *T*. *thermophilus* sequence.

Here, we have solved by X-ray crystallography the structure of methylenetetrahydrofolate dehydrogenase (MTHFD) from *Thermus thermophilus*. This organism is an extreme thermophilic model organism, which has been described as having an optimal growth ranging from 65°C to 72°C and being resistant to maximum temperatures of 85°C [15,16]. MTHFD is a key enzyme in the folate-dependent one carbon metabolism, which is important for the biosynthesis of several amino acids like glycine, alanine and serine, as well as nucleotide bases, formylated methionine and some pro-vitamins [17,18]. In some bacteria like *E*. *coli*, MTHFD (called FolD in this species) is a dual function enzyme that catalyzes the conversion of 5,10‐methylenetetrahydrofolate and NADP^+^ to 5,10-methenyltetrahydrofolate and NADPH (dehydrogenase function), and further converts the first product to 10-formyltetrahdrofolate (cyclohydrolase function) [19] (**Fig 1A**). Some microorganisms have a separate enzyme for catalyzing the second cyclohydrolase function (FchA) [20], and some species have an alternative pathway for 10-formyltetrahdrofolate production using formyltetrahydrofolate synthetase (Fhs) [21]. In eukaryotes, MTHFD is an important anticancer therapy target [22,23].

To analyze possible determinants of *Thermus thermophilus* MTHFD (*TtMTHFD*) thermostability, we have carried out 300 ns MD simulations of this enzyme at three temperatures (29.9°C / 303 K, 59.9°C / 333 K and 89.9°C / 363 K). Additionally, the enzyme was compared with two related prokaryotic enzymes from the thermoacidophilic archaeon *Thermoplasma acidophilum* (PDB identifier 3NGL) [24], which is known to grow at temperatures between 45°C and 62°C with an optimum temperature of 59°C [25,26] and the mesophilic gram-negative opportunistic human pathogenic bacterium *Acinetobacter baumannii* (PDB identifier 4B4U) [27], which thrives between 25°C and 45°C, with maximum growth rates at 37°C [28]. To analyze the obtained MD trajectories, we combined several well-known analysis tools such as Principal Component Analysis (PCA) and Residue Interaction Network (RIN) analysis. For rotamer analysis, we adapted methods from other research fields, like Gini coefficient analysis and Kullback–Leibler (KL) divergence analysis. All these methods, together with clustering methods, provide data and dimension reduction capabilities which we used to identify important amino acids that determine the heat stabilization of the protein.

Principal component analysis (PCA) is a dimensionality-reduction method that can be applied to simplify complex data sets [12]. Briefly, a covariance matrix of the atomic coordinates of a MD trajectory is diagonalized to obtain its eigenvectors and associated eigenvalues, which represent a measure of the main divergence of the original trajectory. Thus, PCA can be used determine the major movements of proteins, which are called, in this sense, ‘principal components’ (PCs).

Residue Interaction Network (RIN) analysis was carried out using the concept of betweenness centrality (BC). Essentially, every amino acid in a protein (as represented by the position of its C_α_ and C_β_ atoms) is considered a node in an interaction network with the edges characterizing node interaction defined when the C_β_ atoms are located within a given cut off distance. The number of the shortest paths between two residues that pass through a chosen node is termed ‘betweenness centrality’ of the node. BC can be considered a measure of the structural prominence of a residue [13].

Gini coefficients, originally introduced in econometrics to describe the equality of income distributions [29], are defined as the ratio between the areas under a true income distribution and a total equality distribution. In our analysis, we borrowed this notion and applied it as a simple measure of the breadth of distribution of side chain rotamers. Side chain rotamers are defined by the usual χ-angle torsion conformations of a protein’s side chains [30]. Smaller Gini coefficients indicate a rotamer distribution tending towards a uniform distribution. Negative differences of the Gini coefficients of rotamer distributions at two different temperatures were interpreted as a measure of entropy-related energy dispersion effects.

Gini coefficient analysis was additionally corroborated using Kullback-Leibler (KL) divergence analysis, which is a finer measure of discrete probability distributions. KL divergence (D_KL_), often called the relative entropy, is defined as

$$D_{KL}(P||q)=\sum P_{i}\log(\frac{P_{i}}{q_{i}})$$ (1)

where *P* and *Q* are two discrete probability distributions satisfying $0<p_{i},q_{i}<1$, $\sum p_{i}=1$ and $\sum Q_{i}=1$. It was originally introduced by Shannon to express the information content of a message transmitted to a receiver [31]. When the sender and receiver probability distributions share similar representations of a message’s information content, D_KL_(P||Q) vanishes. Non-vanishing KL divergence is a qualitative measure of discrimination between sender and receiver information content. Here, we interpret D_KL_(P||Q) to discriminate two side-chain rotamer probability distributions at different temperatures in terms of information content but, more importantly for our purposes, as an indication of entropy related energy dispersion effects.

Finally, to combine these different data reduction methods, dimensionless Z-scores (in form of standard deviations away from the mean) were introduced and clustering techniques applied. Clustering is an unsupervised machine learning technique that groups objects based on their features. Hierarchical clustering is based on distance connectivity [32] and was used here to filter the MD analysis data for important residues involved in the thermostability of TtMTHFD.

## Methods

### Cloning, expression and purification

*T*. *thermophilus* HB8 methylenetetrahydrofolate dehydrogenase (MTHFD, GenBank entry Q5SJ94.1) was amplified via PCR amplification of genomic DNA (DSMZ—German Collection of Microorganisms and Cell Cultures, Braunschweig, Germany) using the DNA oligonucleotides 5’-GGCCGGAGATCTGTGGCGGCCCAGGTGCTTTCGGGACACGAG-3’ and 5’-GGCCGGAAGCTTAGCCAGAAGCTCCATGGCGCCTCAAGAGGG-3’ (Exxtend, Paulínia, Brazil) as primers and inserted into the BamHI and HindIII sites of the pQtev His-tag *E*. *coli* expression vector (Protein Structure Factory, Berlin, Germany). The construct was confirmed by sequencing (Exxtend, Paulínia, Brazil). TtMTHFD was expressed recombinantly in *E*. *coli* BL21(DE3) at 37°C for 18h after induction in LB medium with 1 mM Isopropyl-ß-D-1-thiogalactopyranoside (IPTG). Cells were harvested by centrifugation at 4500 rpm for 15 min and resuspended in lysis buffer consisting of 50 mM Tris–HCl pH 8.0, 200 mM NaCl, 10 mM imidazole, 1 mM PMSF, 5 mM ß-mercaptoethanol, 1% Brij100, 1 mg mL^-1^ Lysozyme and 2 ng mL^-1^ DNAse. The enzyme was purified from the soluble fraction by affinity chromatography as a 33 kDa His-tagged protein using a 5 mL HisTrap Sepharose column (GE Healthcare) on an ÄKTAprime Plus liquid-chromatography system (GE Healthcare). After dialysis in 50 mM Tris–HCl pH 8.0, 200 mM NaCl, 3 mM DTT, the enzyme was concentrated to 20 mg mL^-1^ using Amicon Ultra-15 centrifugal filters (Millipore).

### Crystallization and crystallography

*Tt*MTHFD was crystallized by the hanging-drop method using 1 μL drop/1 μL reservoir ratio conditions in 24-well plates under conditions containing 800mM Potassium Sodium Tartrate and 100 mM HEPES buffer pH 7.5 as crystallization buffer. The obtained crystals were measured after flash-cooling in liquid nitrogen using crystallization buffer supplemented with 26% glycerol. Two datasets at 1.5406 Å and 1.4587 Å were obtained on a Rigaku MicroMax-007 HF microfocus rotating anode diffractometer with an R-AXIS IV++ image plate detector at the Analytical Center at the Chemistry Institute of São Paulo University (IQ-USP, São Paulo, Brazil) and the MX2 Beamline at the Brazilian Synchrotron Light Laboratory (LNLS, Campinas, Brazil) [33]. Crystallographic data were processed with XDS [34]. The structure of MTHFD was solved by molecular replacement (MR) using the Phaser [35] module of PHENIX [36]. Electron-density maps were inspected and the structural model was built using Coot [37].

### Molecular dynamics (MD)

MD simulations were carried out using the AMBER [38] simulation software package. Parametrization of the NADP^+^ nucleotide was performed using antechamber and the semi-empirical AM1 with bond charge correction (AM1-BCC) method [39]. The MTHFD NADP^+^ complexes were processed with tleap using the ff14SB AMBER force field for the protein and the Generalized Amber Force Field (GAFF) for the nucleotide, neutralized with counter-ions and solvated with the TIP3P water model in a cuboid integration box with 14 Å solvent margins from the complexes. After structure minimizations, short heating MD runs and density equilibration MD runs, 300 ns molecular dynamic production runs of all three dimeric complexes were performed using the GPU-version of pmemd at three temperatures (303 K, 333 K and 363 K). Production runs were carried out using 2 fs time steps, constant pressure with isotropic position scaling, Particle Mesh Ewald (PME) periodic boundary conditions with an 8 Å classical non-bonded cut off, a 2 ps collision frequency Langevin thermostat, the SHAKE algorithm with bonds involving hydrogens constrained and bond interactions involving hydrogens omitted. A total of 200.000 snapshots were produced. RMSD, RMSD per-residue and radius of gyration analysis was carried out with cpptraj.

### Principal component analysis

Principal Component (PC) Analysis was carried out to compare and analyze main trajectory divergences between trajectories at two temperatures using cpptraj [40] as described by Galindo-Murillo *et al*. [41]. Basically, both trajectories were concatenated, RMS-fitted to the first frame and an average trajectory calculated that was fitted to all other frames. Next, the covariance matrix between the three coordinates of all C_α_ atoms was calculated and diagonalized to determine its eigenvectors and associated eigenvalues. The coordinates of the trajectories were then projected onto the eigenvectors to determine the influence of residues on the principal components by scalar multiplication p=〈x〉⋅e, where ***x*** are the three vector components of the average position of each atom and ***e*** the corresponding vector components of a specific eigenvector. A mass weighted sum of the *P* values of all atoms in a residue was calculated to obtain each residue’s participation on the specific principal component. Additionally, principal component trajectory interpolations were carried out. Principal component distribution was evaluated by histograms in bins by projecting the C_α_ trajectory coordinates on the corresponding eigenvector components. The obtained values were used to calculate interpolated trajectories, such as P=〈X〉+λE, where 〈x〉 is the average coordinate over the whole vector space and *E* a specific eigenvector and λ evaluated from the histograms.

### Residue Interaction Network (RIN) analysis

RIN analysis was carried out using MD-TASK [13]. Trajectories were reduced to C_α_ and C_β_ atoms using cpptraj. Betweenness centrality (BC) was calculated using the calc_network.py script. Networks at two different temperatures were compared using the compare_networks.py script.

### Rotamer analysis

The rotamer distribution of the amino acid side chains (chip, chi2, chi3, chi4) of the proteins were evaluated using cpptraj based on a library of the most common rotamers in protein structures [42], using the algorithm described by Haddad *et al*. [43]. Differences between the Gini coefficients at different temperatures were calculated using following equation for the Gini coefficients

$$G=\frac{\sum_{i=1}^{n}\sum_{j=1}^{n}\left|x_{i}-x_{j}\right|}{2n^{2}\langle x\rangle },$$ (2)

where *n* is the total number of rotamers of an amino acid and *x*_*i*_ is the observed number of the *i*^th^ rotamer and 〈x〉 is the average value of the rotamer sampled over a trajectory. The KL divergence D_KL_(P||Q) was calculated using the distribution of side-chain rotamers relative to a uniform distribution of those side-chain rotamers.

### Heat maps

For heatmap calculations, sequences were aligned with Clustal Omega [44] and inserts between structures removed after manual inspection. Per-residue PCA component values from temperature difference PCA analysis, Gini coefficient differences, temperature dependent BC analysis values and difference KL (ΔKL) values for two temperature distributions when compared with the corresponding uniform distributions were then loaded into a comparison matrix using Z_i_ scores (based on the ΔKL, GCD, PC1, DBC for the three different temperatures), where $Z_{i}=(x-\langle x\rangle )/sd(x)$. Besides correlation analysis, a hierarchical cluster analysis was performed on the comparison matrix using the Python Seaborn library (https://github.com/mwaskom/seaborn/tree/v0.8.1). Additionally, an overall per-residue Z-score was calculated as $z=\sqrt{\sum z_{i}^{2}}$.

### Residue identity histograms

Residues with similar composite Z-scores of a protein were binned. Then, the average amino acid identity of these binned amino acids calculated in comparison with one of the other proteins. Finally, the calculated amino acid identity values were plotted over the Z-score bins.

### Graphical representations

Protein structures were rendered with PyMOL [45]. Molecular structures were drawn with ChemSketch (Advanced Chemistry Development, Inc., Toronto). Graphs were drawn using gnuplot (http://gnuplot.sourceforge.net/) or matplotlib [46]. For visualization of rotamers, their MD trajectory rotamer library values were clustered after standardization and PCA analysis using the k-means clustering algorithm with the Scikit-learn python machine learning library as described in https://medium.com/@dmitriy.kavyazin/principal-component-analysis-and-k-means-clustering-to-visualize-a-high-dimensional-dataset-577b2a7a5fe2.

## Results and discussion

### The MTHFD fold

In order to gain more insight into the temperature resistance of *T*. *thermophilus* MTHFD (TtMTHFD), we produced the enzyme recombinantly in *E*. *coli* BL21(DE3), purified and crystallized the protein. The crystal structure was solved at 2.15 Å by molecular replacement using the 3P2O PDB entry (MTHFD from *Campylobacter jejuni*) as a search model. The final model included one chain of TtMTHFD, one NADP^+^ nucleotide molecule and 60 water molecules in the asymmetric unit. The model can be described after refinement by a crystallographic R factor of 21.3% with a corresponding R_free_ of 24.4% (**Table 1**). TtMTHFD consists of two α/β-fold sub-domains connected by two large α-helices, which are at the same time the N-terminal and C-terminal α-helices. The N-terminal α/β-fold sub-domain is the catalytic domain that binds the main substrate, and the C-terminal α/β-fold sub-domain is the nucleotide (co-substrate) binding domain, which are variants of the structurally conserved Rossmann dinucleotide-binding domain fold (**Fig 1B**). Both domains are interconnected by an extended loop-helix-loop motif that we here termed ‘Connector Helix (CH) region’. In the nucleotide binding domain, we were able to unambiguously identify and fit a molecule of NADP^+^ as shown in **Fig 1C**. The obtained structure showed, as expected, a high degree of conservation when compared to similar bacterial MTHFD structures (**Fig 1D** and **1E**). In order to compare TtMTHFD, we chose two related MTHFD structures from *Thermoplasma acidophilum*, an organism with growth optimum between 45°C and 62°C (PDB entry 3NGL) and *Acinetobacter baumannii*, an organism with growth optimum between 25°C and 45°C (PDB entry 4B4U). Compared to the *Thermoplasma acidophilum* MTHFD (TaMTHFD) and *Acinetobacter baumannii* MTHFD (AbMTHFD) structures, *T*. *thermophilus* MTHFD (TtMTHFD) showed an overall RMSD of 0.98 Å (with an overall sequence identity of 36%) and an overall RMSD of 1.29 Å (with an overall sequence identity of 39%), respectively (**Fig 1D**).

**Table 1:** Crystallographic data collection and structure refinement statistics.^a^

**Crystallographic data collection statistics**	
Diffraction source	MX‐2 beamline, LNLS
Wavelength (Å)	1.4587
Temperature (K)	100
Detector	PILATUS2M
Crystal‐detector distance (mm)	205.12
Rotation range per image (°)	0.1
Data range	1–1800
Space group	P3_2_21
*a*, *b*, *c* (Å)	121.36 121.36 59.62
α, β, γ (°)	90.0 90.0 120.0
Mosaicity (°)	0.221
Resolution range (Å)	39.42–2.152 (2.229–2.152)
Total number of reflections	209550 (6228)
Number of unique reflections	25688 (1122)
Completeness (%)	92.32 (38.23)
Redundancy	8.53 (2.96)
〈I/σ(I)〉^b^	19.87 (0.85)
*R*_*meas*_ (%) ^c^	8.7 (143.1)
*CC*_1/2_ ^d^	99.9 (43.3)
Resolution range (Å)	39.42–2.152 (2.229–2.152)
Completeness (%)	92.32 (38.23)
Number of reflections, test set	25581, 1283 (1044, 55)
**Refinement statistics**	
*R*_*work*_ Final ^e^	0.2131 (0.3860)
*R*_*free*_ Final ^f^	0.2439 (0.4540)
Number of nonhydrogen atoms	2235
Protein residues	277
Water	60
RMSD^g^	
Bonds (Å)	0.008
Angles (°)	0.95
Average *B*‐factors (Å^2^)	51.13
Ramachandran plot	
Most favored (%)	97.45
Allowed (%)	2.55
Outliers (%)	0.00

^a ^Values for the highest-resolution shell are shown in parentheses.

^b^ Signal-to-noise ratio.

^c $R_{meas}=\Sigma_{hkl}{\left(n/\left(n-1\right)\right)}^{1/2}\Sigma_{i}\left|I_{hkl,i}-\left\langle I_{hkl}\right\rangle \right|\Sigma_{hkl}\Sigma_{i}I_{hkl,i}$^, for *n* symmetry related refection intensities *I*_*hkl*_,_*i*_.

^d^ *CC*_1/2_ Pearson’s correlation coefficient calculated with data set randomly split in half.

^e^ *R*_*work*_^$=\sum \left|F_{o}\vee -F_{c}\right|\vee \sum F_{o}\vee$^, where *F*_*o*_ *V*and *F*_*c*_ *V*are the observed and calculated structure factor amplitudes.

^f^ *R*_*free*_ was calculated as *R*_*work*_ with 10% of the data omitted from structure refinement.

^g^ RMSD., root mean square deviations from ideal geometry.

Concerning salt bridges, both TtMTHFD and TaMTHFD have 67 charged surface amino acids, while AbMTHFD has 57 charged surface amino acids. Conversely, using a cut-off value of 4 Å, a higher amount of salt-bridges in the crystal structure of TtMTHFD (n = 26), when compared with TaMTHFD (n = 16) and AbMTHFD (n = 10), could be identified. Interestingly, again TtMTHFD showed a higher number of inter-chain salt bridges (n = 6) in comparison with TaMTHFD (n = 2) and AbMTHFD (none), indicating the importance of salt bridges for the thermo-stabilization of dimers. Furthermore, TtMTHFD had a higher number of arginine-formed salt-bridges (n = 23) compared with TaMTHFD (n = 5) and AbMTHFD (n = 6), as expected for thermophilic proteins [47].

**Fig 2:**
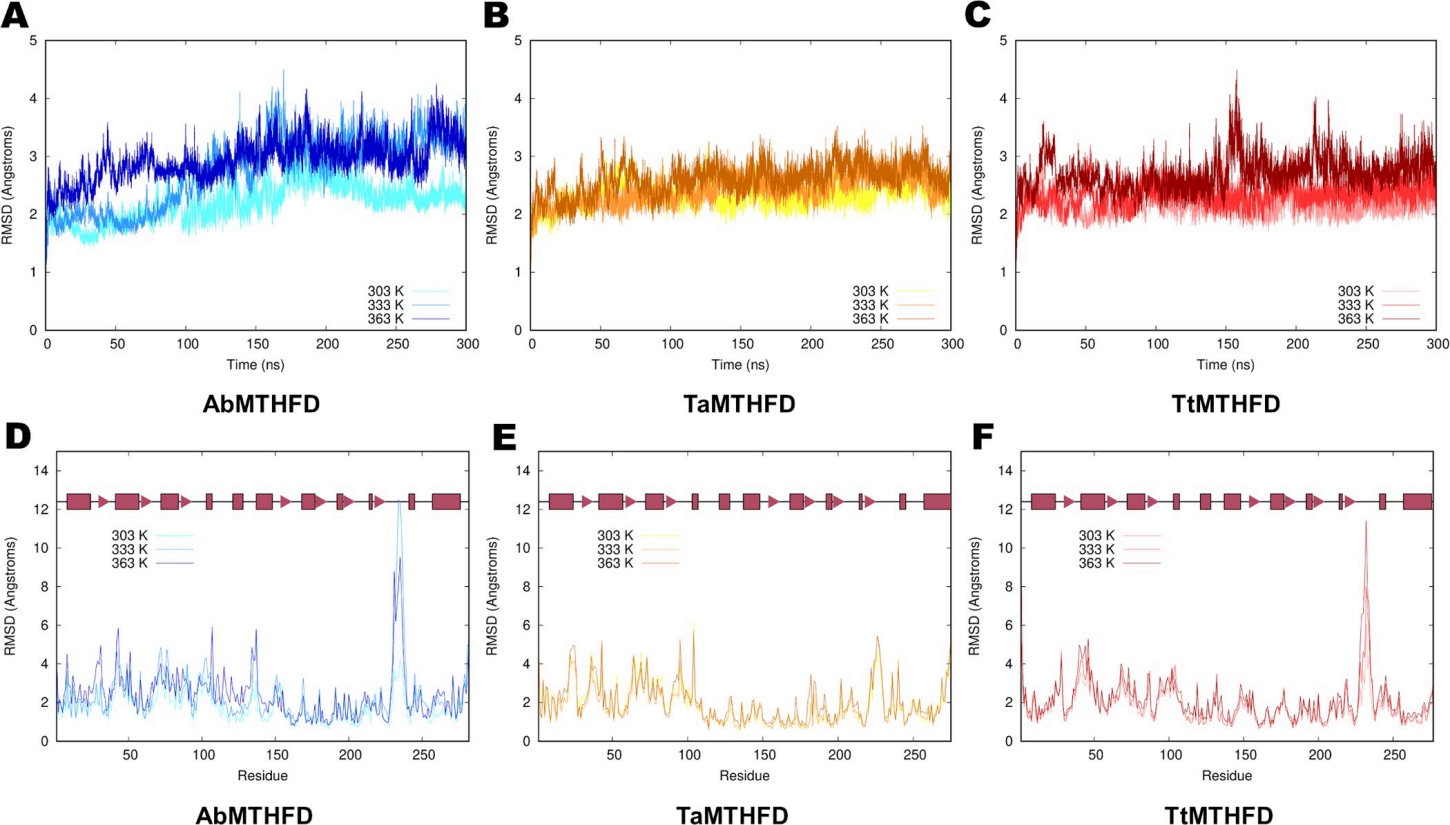
RMSD trajectory analysis. Comparison of RMSD plots (in Å) of C_α_ atoms of 300 ns MD simulations of (A) *A*. *baumannii* at different temperatures (303 K light blue, 333 K blue, 363 K dark blue), (B) *T*. *acidophilum* (303 K yellow, 333K light orange, 363 K dark orange) and (C) *T*. *thermophilus* MTHFD (303 K light red, 333K red, 363 K dark red). Comparison of per-residue average C_α_ RMSD values (in Å) of (D) *A*. *baumannii* (E) *T*. *acidophilum* and (F) *T*. *thermophilus* MTHFD with colors as shown above and secondary structure elements indicated in red (boxes for α-helices, triangles for β-sheet strands).

As crystal structures measured under cryo-conditions give a static view of proteins, 300 ns molecular dynamics (MD) simulations of the dimeric form of all three proteins where carried out at three temperatures (303 K, 333 K and 363 K). These temperatures partially represent the appropriate temperatures for mesophilic organisms, like *A*. *baumannii*, a thermophilic organism like *T*. *acidophilum* and an even more thermoresistant organism like *T*. *thermophilu*s. The corresponding RMSD values over the trajectories of these simulations are shown in **Fig 2A, 2B** and **2C** and the corresponding per-residue average RMSD values of all non-hydrogen atoms are shown in **Fig 2D, 2E** and **2F**. Besides small trends, we could not detect significant shifts of RMSD values (**Fig 2A, 2B** and **2C**) nor the radii of gyration. The radius of gyration of the monomers oscillated at the three temperatures around an average of 19.3 Å ± 0.2 Å for AbMTHFD, 19.1 Å ± 0.2 Å for TaMTHFD and 19.4 Å ± 0.2 Å for TtMTHFD.

Regarding the RMSD values over time (**Fig 2A, 2B** and **2C**), a slight trend to higher RMSD values with temperature could be seen in all three proteins. This is especially true for the mesophilic *A*. *baumannii* structure where a separation of the RMSD values could be seen, indicating a possible partial denaturation of the protein with higher temperatures. Moreover, the per-residue RMSD (**Fig 2D, 2E** and **2F**) showed a similar distribution in all three structures. In this analysis, a high correlation of RMSD values between the residues in each monomer of the simulated dimers could be observed. Pearson correlation coefficients ranged from 0.75 to 0.96 with an average value of 0.90, indicating a coherent behavior of the MD simulations. Higher RMSD values correlated with loop regions, as expected. All three proteins showed similar distributions of RMSD values, with the notable exception of the *T*. *acidophilum* lid loop region, which showed a slightly lower RMSD values when compared to the homologous regions of the other two structures (**Fig 2D, 2E** and **2F**).

### Principal component analysis

Because molecular dynamics is a thermodynamic sampling technique, we hypothesized that a more systemic variation technique like principal component analysis (PCA) could be deployed to extract the main distinctions in movement at different temperatures. Thus, to obtain more insight on possible underlying mechanisms behind thermo-resistance, PCA was carried out so that two temperatures were compared, e.g. comparing 303 K to 363 K trajectories.

**Fig 3:**
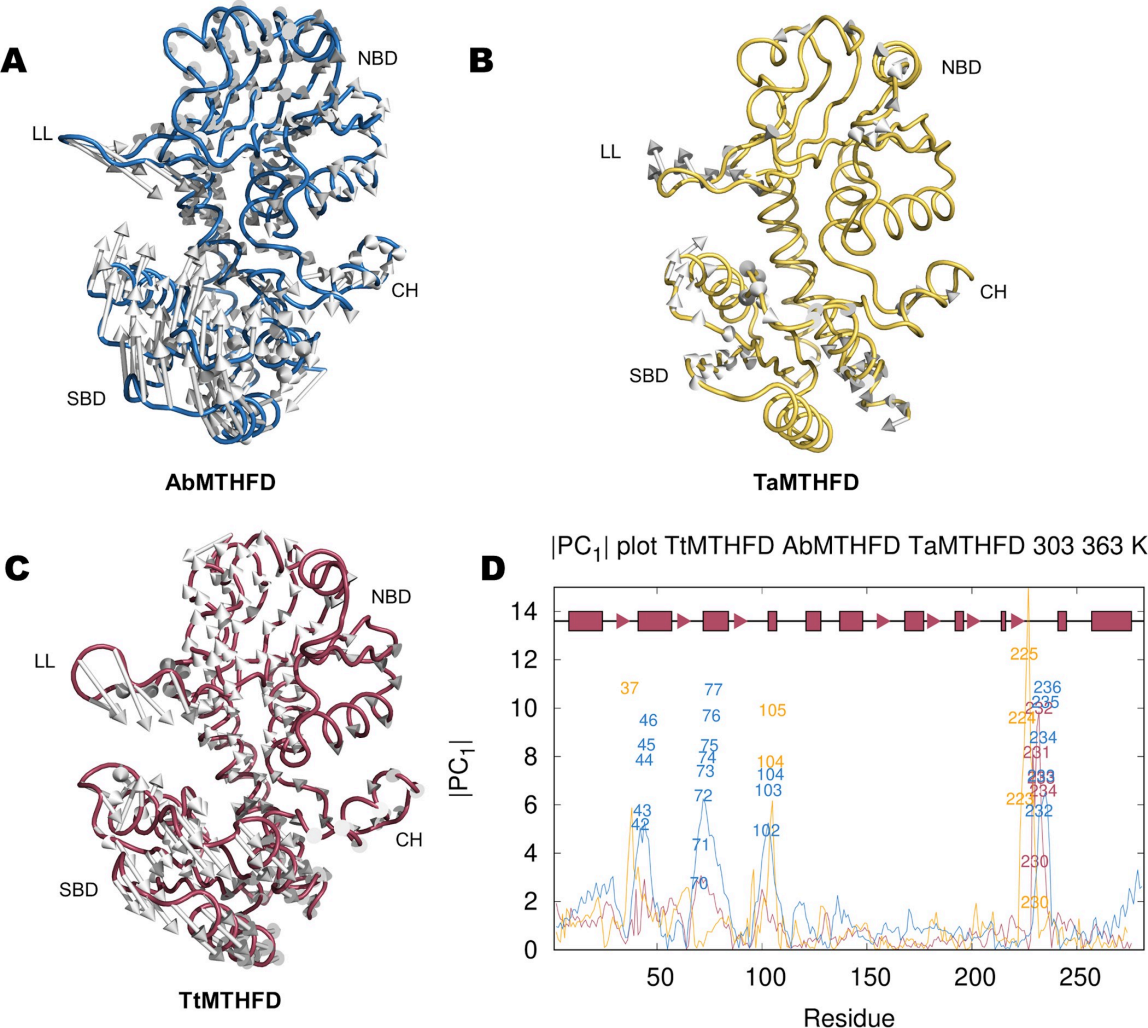
Principal component analysis. Comparison of 1^st^ PC eigenvector component interpolations of C_α_ atoms of (A) *A*. *baumannii* (blue), (B) *T*. *acidophilum* (yellow) and (C) *T*. *thermophilus* (red) MTHFD trajectories formed by concatenation of 300 ns of the 303 K and 363 K MD simulations indicates major characteristic 1^st^ PC movements/deviations of substrate binding domain and lid loop. (D) Comparison of amplitudes of first principal components (|*PC*_1_|) for each residue of the three simulated proteins, colored as described above, showing important regions with high PC deviations, e.g. in TtMTHFD the lid loop (amino acids 226 to 239), substrate binding domain (amino acids 26 to 107) and connector helix region (amino acids 108 to 136). Important amino acids of each protein, selected by applying a 2 standard deviations over average value cut-off, are shown in the respective colors described above. In TtMTHFD lid loop amino acids Arg 230, Val 231, Glu 232, Gly 233 and Arg 234 show elevated 1^st^ PC values. In TaMTHFD the elements with high 1^st^ PC values are found on residues of the lid loop (Ile 223, Asn 224, Tyr 225, Val 230). Additionally, residues on structures opposite to the lid loop (e.g. Asp 37) and on the α4 helix (Val 104 and Arg 105), which is adjacent to the connector helix, show elevated 1^st^ PC values. In the case of AbMTHFD, numerous amino acids show elevated 1^st^ PC values. These amino acids belong to either the lid loop (His 232, Pro 233, Arg 234, Asp 235, Gly 236) or the adjacent loops from the substrate binding domain (Asp 42, Asp 43, Gly 44, Ala 45, Ser 46; Pro 70, Gln 71, Glu 72, Thr 73, Thr 74, Thr 75, Glu 76, Gln 77; Ala 102, Gln 103, Ile 104).

Important insight can be gained by interpolating movements along the eigenvectors of the PCs (**Fig 3A, 3B** and **3C**). Several types of movements could be identified. The most prominent movements were: opening/closing of the lid loop and different directed movements leading to opening/closing or distortion of the substrate binding domain. In the case of TtMTHFD, displacements of C_α_ atoms between 303 K and 363 K showed precisely these two movements. Firstly, the lid loop closes downwards in direction of the substrate binding site. Secondly, the substrate binding domains followed a well-defined movement (1^st^ PC, eigenvalue of 295, 2^nd^ PC, with a similar eigenvalue of 231). These movements evolve around hinges on the N-terminal helix and the region of the connector helix. Similar patterns could be detected in the PCA of the 333 K to 363 K and 303 K to 333 K trajectories of TtMTHFD (not shown). Altogether this indicates that TtMTHFD shows similar coordinated movements at different temperature steps. In the case of AbMTHFD, the movements are similar. In the case of TaMTHFD, the movements of the substrate binding domain look overall less coordinated, implying possibly more distortions within this domain.

These main movements described here, are possibly a structural characteristic that is intrinsic to the dual α/β-domain fold of MTHFD, as similar movements were deduced by simply comparing the two monomers in the dimer of previously described crystal structures [17,18]. In these dimeric crystal structures, superposition of the NCS related monomers show a torsion of the substrate binding domain at ‘hinges’ on the N-terminal and α-helical connecting domains, thus in regions very similar to the ones identified by our PCA. Therefore, overall, PCA analysis confirms qualitatively the expected motion of the substrate binding domain.

To expand the overall motion to the residue level, trajectory projections were calculated. In detail, these trajectory projections consist of multiplying the average coordinate of the C_α_ atoms of the trajectories by the corresponding components of the eigenvectors to obtain the absolute value of the contribution of each C_α_ to the 1^st^ PC (**Fig 3D**). The most prominent structural element showing high 1^st^ PC values in TtMTHFD, TaMTHFD and AbMTHFD includes the lid loop. Additionally, some of the loops from the substrate binding domain show elevated 1^st^ PC values, especially in TaMTHFD and mostly prominently in AbMTHFD. These less thermoresistant proteins consequently showed more regions with elevated 1^st^ PC values. It is important to add that the average Pearson correlation coefficient of all trajectory projections between both monomer sub-units of the dimer concerning the 1^st^ PC was 0.48.

In conclusion, PCA highlighted the main differences between the protein at different temperatures and could be described at the amino acid level using the vector projections. Many of these displacements showed a similar trend at different temperatures, indicating that PC movements are qualitatively based on the structural movements of the protein fold. We therefore set out to determine other important descriptions of differences in temperature that could be linked to the observations derived from PCA.

### Residue interaction networks

**Fig 4:**
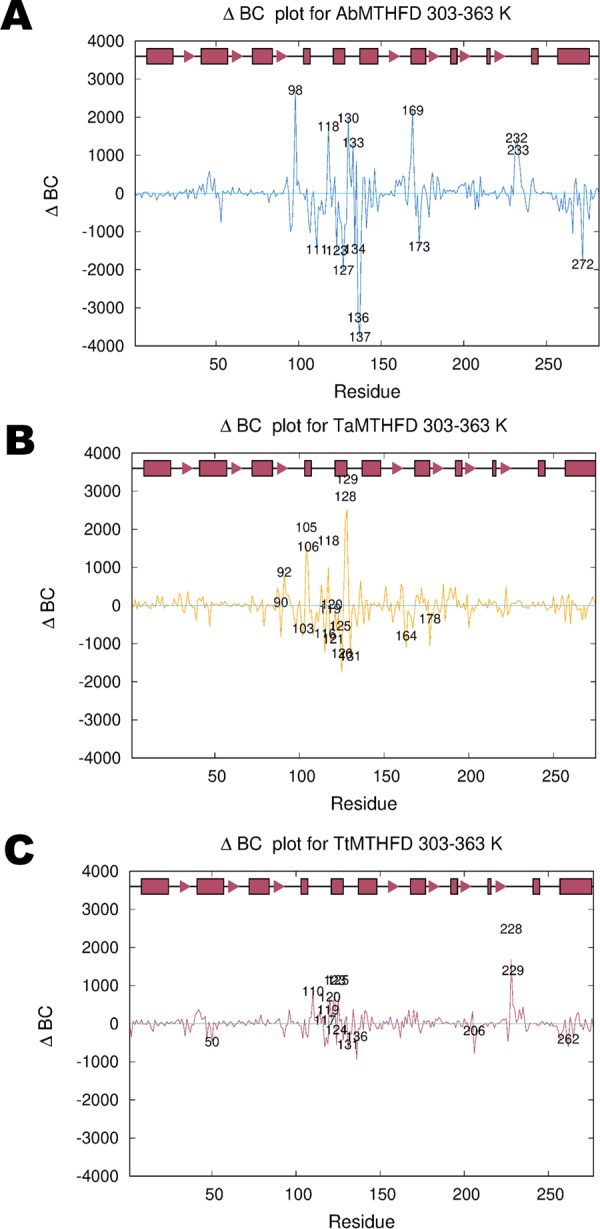
Residue interaction network analysis. Comparison of per-residue betweenness centrality (BC) differences (ΔBC) of C_α_/C_β_ atoms of 303 K/363 K trajectories of (A) *A*. *baumannii* (blue), (B) *T*. *acidophilum* (yellow) and (C) *T*. *thermophilus* (red) molecular dynamics simulations showing important amino acids involved in fold stabilization with important residues highlighted based on a cut-off value of 2 standard deviations. These amino acids cluster mostly around the connector helix region in all three structures. (A) AbMTHFD shows significantly increased BC values in several residues that make up the interface between the substrate binding, co-factor binding domain, connector helix region and lid loop (e.g. His 98, Asp 118, Met 130, Gly 133, Ile 169, His 232 and Pro 233). The amino acids with the greatest decrease in BC values are Phe 127 and Tyr 137 (together with Ala 136). The decrease in BC for these bulky amino acids could indicate the onset of denaturation. (B) TaMTHFD shows a similar but stronger distribution of residues with higher BC values when compared with TtMTHFD (e.g. Arg 105, Asn 106, Pro 118, Arg 128 and Glu 129). All these residues are centered on the connector helix region of the protein. (C) In TtMTHFD, the connector helix showed elevated betweenness centrality (BC) for several of its residues, specifically His 120, Asn 123 and Gly 125. Additionally, the hydrophobic residues from the lid loop region, Val 228 and Asn 229, showed significantly increased BC values.

As PCA showed important conformational changes, we then carried out residue interaction network analysis (RIN) on the trajectories of the three proteins (**Fig 4A, 4B** and **4C**). Again, these images showed subtle but detectable differences between the three proteins, this time centered around the connector helix region. Like in the case of 1st PC projections, also RIN analysis showed less movement for the more thermostable protein TtMTHFD. As a whole, the BC distribution of amino acids affected by temperature jumps were similar in all three structures. AbMTHFD showed a movement that could be interpreted as partial denaturation. TaMTHFD showed a border-line behavior between TtMTHFD and AbMTHFD. Regarding BC differences, the more thermophilic TtMTHFD showed smaller movements reminiscent of a more thermo-resistant protein. This allows to narrow down the analysis of movements to obtain a more mechanistic view of the temperature effects. However, the interpretation of both the PC and RIN analysis could be more related to effects than causes. Therefore, a more causative explanation of the observed displacements was required.

### Rotamers

**Fig 5:**
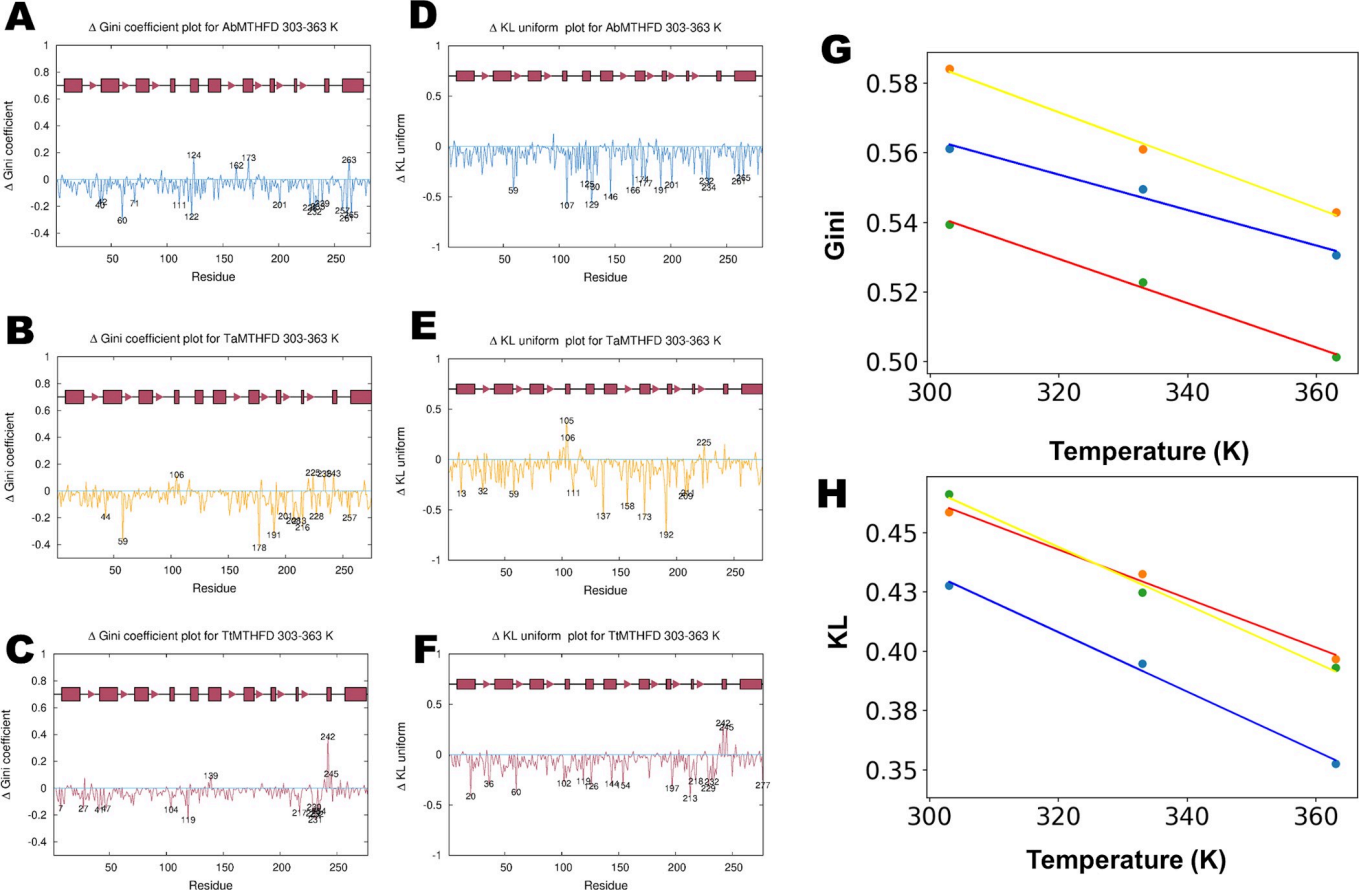
Rotamer analysis. Per-residue rotamer distributions plotted as Gini coefficient differences (ΔGini, GCD) calculated for the 303 K and 363 K trajectories of the (A) *A*. *baumannii* (blue), (B) *T*. *acidophilum* (yellow) and (C) *T*. *thermophilus* (red) molecular dynamics simulations. Additionally, the ΔKL (difference Kullback-Leibler divergence) coefficients were calculated in a similar manner for the (D) *A*. *baumannii*, (E) *T*. *acidophilum* and (F) *T*. *thermophilus* simulations. Important residues with a GCD and ΔKL cut off above 2 standard deviations, are highlighted showing a more uniform distribution in the more thermophilic *T*. *thermophilus* structure than the other two proteins. (A) AbMTHFD shows several amino acids with significantly reduced Gini coefficients. Whereas some residues are solvent exposed (His 232, Asp 235, Val 239, on lid loop; Asp 42, Glu 111, Gln 201, Asp 228 on other regions), several of these amino acids are in the hydrophobic core (Val 40, Val 60, Val 122, Val 257, Val 261, Thr 265), confirming an indication of a beginning denaturation of the protein. (B) TaMTHFD showed less residues with strongly reduced Gini coefficients (like Tyr 44, Val 59, Ser 178, Thr 191, Val 201, Gln 208, Thr 213, Ser 216, Asp 228, Thr 257) during the 303 K to 363 K transition. (C) TtMTHFD showed several surface-exposed mostly hydrophilic and charged amino acids with significantly decreased values (e.g. Asn 229, Val 231, Glu 232 and Arg 234 from the lid loop, as well as Ser 7, Ser 27 and Gln 104 from other regions). Additionally, some hydrophobic side-chain residues like Pro 41 and Phe 119 as well as hydrophobic core related residues like Val 47 and Val 217 showed significantly decreased Gini coefficients. (D) Similar to the Gini analysis, AbMTHFD showed significantly lowered KL divergence for both solvent exposed and buried amino acids, like His 232, Arg 234 (lid loop), Arg 166, Arg 191, Gln 201 (solvent exposed, nucleotide binding domain), Met 146, Met 174, Met 177 (buried, nucleotide binding domain) Leu 125, Arg 129, (connector helix, solvent exposed), Met 130 (CH, buried), Val 261, Thr 265 (C-terminal helix); Arg 59 and Arg 107 (nucleotide binding domain). (E) TaMTHFD showed significantly lowered KL divergence for the solvent exposed amino acids Lys111, Arg 137, Arg 158, Arg 173, Arg 192, Arg 209 and the semi exposed residue Met 211 (from the nucleotide binding domain), Lys 13 (solvent exposed, N-terminal helix), Leu 32 and Val 59 (buried, substrate binding domain). (F) In TtMTHFD several residues showed significantly lowered KL divergence like Asn 229, Glu 232 (from the lid loop); Arg 144, Arg 197, Arg 213, Arg 218 (solvent exposed charged amino acids from the nucleotide binding domain); Phe 119, Arg 126 (from the CH helix) and Arg 20, Arg 36, Arg 60, Arg 102 (all solvent exposed from the substrate binding domain). (G) Shows the average Gini coefficient values for the three proteins colored in the scheme described above for the three temperatures, indicating that this parameter drops with higher temperatures as expected. (H) To corroborate this, the KL divergence values were plotted for the three temperatures and three proteins.

To obtain more causative insight, we decided to undertake a rotamer analysis of the MD trajectories. This was initially motivated by the hypothesis that charged residues play a role in dynamic thermostability by acting as energy dissipation structures [7,8]. Analysis of side chain conformations of amino acids carried out in crystal structures show a preference for a limited number of the so called rotamer conformations. Thus, a published rotamer library was used to classify the rotamers in the MD trajectories onto one of these conformations. Next, to describe rotamer distributions, Gini coefficients were introduced as a measure of these distributions. Gini coefficients are normally used in econometrics to measure the inequality (higher Gini values) of income distributions. These Gini coefficients of the conformation distribution among the main rotamers were calculated for the MD trajectories and compared at different temperatures for each protein. For the sake of simplicity, in the following we will refer to this ‘rotamer Gini coefficient’ simply as the Gini coefficient. To detect essential amino acids with a particularly high differences in Gini coefficients, residue plots were constructed (**Fig 5A, 5B** and **5C**).

Because of the simplicity of Gini coefficients, we extended this analysis using the concept of KL divergence. In computational science, KL divergence is an important measure of comparisons between probability distributions. We thus calculated the KL divergence for different temperatures in reference to a uniform distribution of rotamer probabilities, so as to corroborate possible energy dissipation in rotamers (**Fig 5D, 5E** and **5F**). Because of the uniform distribution reference, reduced KL divergence values indicate a more uniform distribution.

As expected, in TtMTHFD most residues show a reduction in both KL divergence and Gini coefficients upon transition from 303 K to 363 K (**Fig 5C** and **5F**). Interestingly, in these figures, the more thermo stable TtMTHFD shows a slightly more uniform distributed peaks with lower intensities, both for the Gini coefficients and KL divergence values. In addition, several charged residues and hydrophobic residues appear to have especially reduced Gini coefficients and KL divergences. This is particularly striking for the KL divergencies of TtMTHFD, where most significant peaks are from charged residues (**Fig 5F)**. Thus, the rotamer analysis confirms the hypothesis that temperature resistant proteins have more charged large amino acids like arginine and lysins on their solvent exposed surface possibly because these residues act like heat energy dissipators. To corroborate the validity of Gini and KL analysis, average Gini and KL values were plotted against the temperature (**Fig 5G** and **5H**, respectively). As can be seen, both average Gini and KL values are reduced with higher temperatures, corroborating their validity.

### Determinants of thermostability

**Fig 6:**
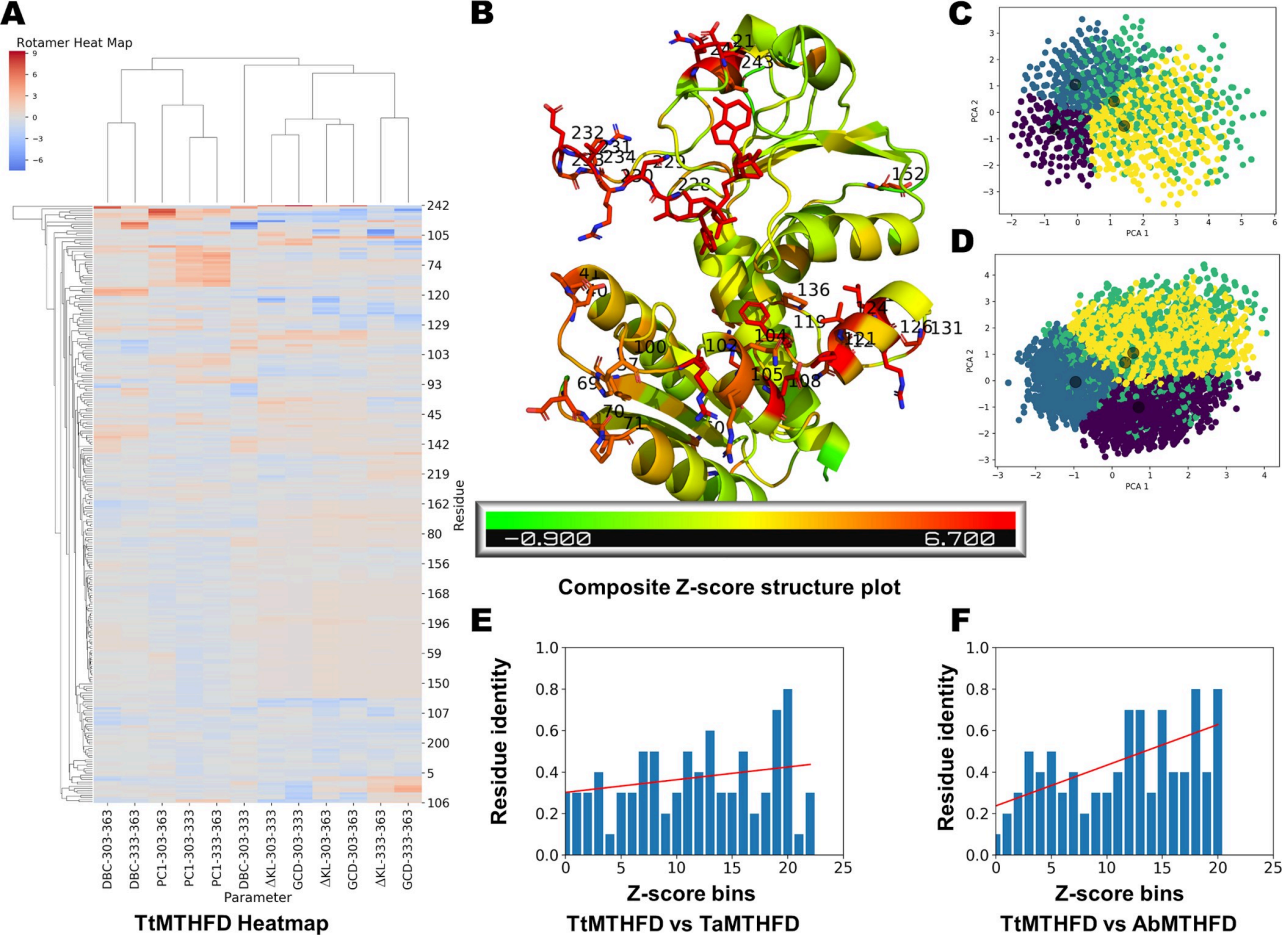
Amino acid heatmap. (A) Obtained per-residue first principal component projections (PC1), betweenness centrality differences (DBC), Gini coefficient of rotamer distribution differences (GCD) and KL coefficient of rotamer distributions differences (ΔKL) Z-scores at the three different temperatures (303 K, 333 K, 363 K as indicated) of the MD simulations of *T*. *thermophilus* MTHFD were hierarchically clustered. While ΔKL values clustered to corresponding GCD values, PC1 and DBC values clustered separately. (B) Clustering allowed to score residues with a composite Z-score and important residues (1 standard deviation above average) are shown projected on the *T*. *thermophilus T*. *thermophilus* MTHFD *T*. *thermophilus* MTHFD MTHFD crystal structure (Gln 104, Glu 108, Pro 121, Asn 123, Val 124, Arg 126, Leu 127, Gly 131, Val 228, Asn 229, Val 231, Glu 232, Glu 245 and Phe 248). PCA of the rotamer library values of the 2 sigma level composite Z-score charged residues of *T*. *thermophilus* MTHFD MD trajectories is shown at 303 K (C) and 363 K (D) demonstrating visually energy dispersion effects. The coloring indicates k-means clustering of the rotamer vectors, which led to an identification of 4 rotamer conformation clusters. Residue identity histograms comparing *T*. *thermophilus* MTHFD with *A*. *baumannii* MTHFD (E) and with *T*. *acidophilum* MTHFD (F) showing that residues with high composite Z-scores (decreasing in bins from left to right) are less conserved. This indicates a selective evolutionary pressure in thermophiles, especially as demonstrated by the TtMTHFD versus TaMTHFD comparison.

Taken together all four analyses indicated important determinants of thermostability in MTHFD. To obtain more mechanistic insight, it is however essential to integrate the different approaches in order to identify correlations between the different descriptors. To scale these ΔKL, GCD, PC1 and DBC values at the three temperatures for comparison, different Z_i_ scores (that is number of standard deviations from the mean) were calculated for these per-residue values. Then, heat map analysis was carried out with the different per-residue Z_i_ scores using hierarchical clustering (**Fig 6A**). The clustering showed strong correlations between the difference KL values (ΔKL) and the Gini coefficient differences (GCD). Additionally, the 1^st^ PC values (PC1) at different temperatures tended to correlate with each other, as did the ΔBC values (DBC).

To highlight better the clustering of residues, composite per-residue Z-score, defined as the RMS of the individual Z_i,_ were calculated. When plotted on the structure, these residues with high composite Z-scores are mostly of two kinds. They are mostly charged solvent exposed residues or semi-buried hydrophobic residues (**Fig 6B**). As examples of these high composite Z-score residues can be cited: Val 228, Asn 229, Val 231 and Glu 232 from the lid loop; Arg 213, Glu 242, Glu 245 from the nucleotide binding domain; Glu 108, Phe 119, Pro 121, Arg 126, from the connector helix region; Arg 102 from the substrate binding domain. Of these cited 12 residues, four are identically conserved in TaMTHFD and two in AbMTHFD. Except the charged residues from the lid loop, all other charged residues from TtMTHFD mentioned form salt-bridges. Thus, some of the residues with high composite Z-scores indeed are involved in salt bridges, which appear to be one of the determinants of thermo stabilization of TtMTHFD. Residues involved in salt bridges in this structure, have an average composite Z-score of 3.8, which can be considered below 1 standard deviation (1.9) above the average composite Z-score (2.9), indicating that not all salt bridges have elevated Z-scores. Regarding the ΔKL value from the 303 K to 363 K jump, charged amino acids of TtMTHFD not involved in salt bridges have a mean value of -0.14, whereas charged amino acids in salt bridges have a value of -0.19. This is an interesting result, as it indicates that salt bridges have an elevated capacity to absorb heat by energy dissipation. Thus, an important conclusion is that in TtMTHFD a large amount of salt bridges probably contributes both statically, i.e. through more elevated Coulomb interactions, as well as dynamically, through higher energy dissipation capabilities, to the stabilization of the protein.

Because it can be assumed that residues with high composite Z-scores probably are structurally important for thermostability, average Pearson correlation coefficients for the different evaluated parameters were calculated. These Pearson correlation coefficients are shown in **Table 2**for all residues of TtMTHFD. Interestingly, a high correlation was found between GCD and ΔKL (0.68) and a slight correlation between PC1 and GCD (-0.37). However, when the residues with high composite Z-score (i.e. with more than two standard deviations from the average) were compared, more significant correlations for GCD/ΔKL (0.74) and PC1/GCD (-0.50) (**Table 3**) could be obtained. Thus, a high composite Z-score seems to indicate amino acids which show elevated correlating behavior between principal components and rotamer energy dispersion. The (negative) correlation between PC1 and GCD is particularly intriguing because it indicates that movement (as described by the PC1 calculated from joint 303 K and 363K trajectories) actually “transfers” energy to rotamers movements during temperature increases. To visualize rotamer energy dispersion, PCA-analysis of the rotamer library value changes at different temperatures during the MD trajectories was carried out. **Fig 6C** shows PCA of the charged residues with 2 sigma level composite Z-scores at 303 K and **Fig 6D** shows PCA at 363 K. From these PCA-projections, the energy dispersion effect of the charged residues becomes apparent, corroborating the notion that charged residue networks could be of great importance for the thermostabilization of proteins.

**Table 2:** Pearson correlation coefficients of evaluated parameters for all amino acids of TtMTHFD.

	PC1-303-363	DBC-303-363	GCD-303-363	ΔKL-303-363
PC1-303-363	1	0.16	-0.37	-0.24
DBC-303-363		1	0.03	0.06
GCD-303-363			1	0.68
ΔKL-303-363				1

**Table 3:** Pearson correlation coefficients of evaluated parameters for amino acids of TtMTHFD with a composite Z-score higher than 2 standard deviations (2 σ level).

	PC1-303-363	DBC-303-363	GCD-303-363	ΔKL-303-363
PC1-303-363	1	0.02	-0.50	-0.26
DBC-303-363		1	0.02	0.10
GCD-303-363			1	0.74
ΔKL-303-363				1

To check if there is any correlation of composite Z-scores with amino acid mutations between TtMTHFD and the other two proteins, residue identity histograms comparing proteins were generated. Indeed, there was a trend of amino acids with high composite Z-scores to be more mutated between TtMTHFD and TaMTHFD (**Fig 6E**). This trend was even more significant when TtMTHFD was compared with mesophilic AbMTHFD (**Fig 6F**). In the case of heat-maps and residue identity histograms for TaMTHFD and AbMTHFD, similar results could be found (**S1A** and **S1B Fig**).

As an overall conclusion, this indicates that composite Z-scores like the one defined in this work, could be considered important descriptors of thermostability in proteins. Thus, composite Z-scores integrating rotamer analysis by KL divergence and Gini coefficients, PC and RIN analysis data from MD simulations can be used to define amino acids that play a more active role in thermostability of protein folding. Furthermore, this type of analysis can eventually be used to predict the effect of mutations on the thermostability of proteins and thus be of help in the protein design of thermostable mutants. We therefore propose an analytical mechanism to detect important mutated residues that determine the thermostability of proteins. It is tempting to speculate, as postulated by Ladenstein *et al*. [7], that rotamer energy dispersion at higher temperatures is causally related to thermostability in proteins like TtMTHFD via a mechanism involving interacting charged residues, as we showed here with help of rotamer distribution analysis. Further research has to be carried out to corroborate and expand this hypothesis.

## Supporting information

S1 Fig Amino acid heat-map of *T*. *acidophilum* MTHFD. (A) PC1, DBC, GCD and ΔKL values were hierarchically clustered for the *T*. *acidophilum* MTHFD MD simulations at the indicated temperatures. Residue identity histograms comparing *T*. *acidophilum* MTHFD with *T*. *thermophilus* MTHFD (B) and with *A*. *baumannii* MTHFD (C). The less thermophilic TaMTHFD showed only a small trend to have more substituted amino acids at high composite Z-score positions when compared with TtMTHFD and had a more significant trend when compared with AbMTHFD.
(TIF)

S2 Fig Amino acid heat-map of *A*. *baumannii* MTHFD. (A) PC1, DBC, GCD and ΔKL values were hierarchically clustered for the *A*. *baumannii* MTHFD MD simulations at the indicated temperatures. Residue identity histograms comparing *A*. *baumannii* MTHFD with *T*. *thermophilus* MTHFD (B) and with *T*. *acidophilum* MTHFD (C). AbMTHFD showed a trend to have more substituted amino acids at high composite Z-score positions when compared with TtMTHFD and had a slight trend when compared to TaMTHFD.
(TIF)
