# Supplementary figures and images for: Crystal structure of Thermus thermophilus methylenetetrahydrofolate dehydrogenase and determinants of thermostability
Source: PLoS One. 2020 May 13;15(5):e0232959. doi: 10.1371/journal.pone.0232959 (PMC7219735; doi:10.1371/journal.pone.0232959)

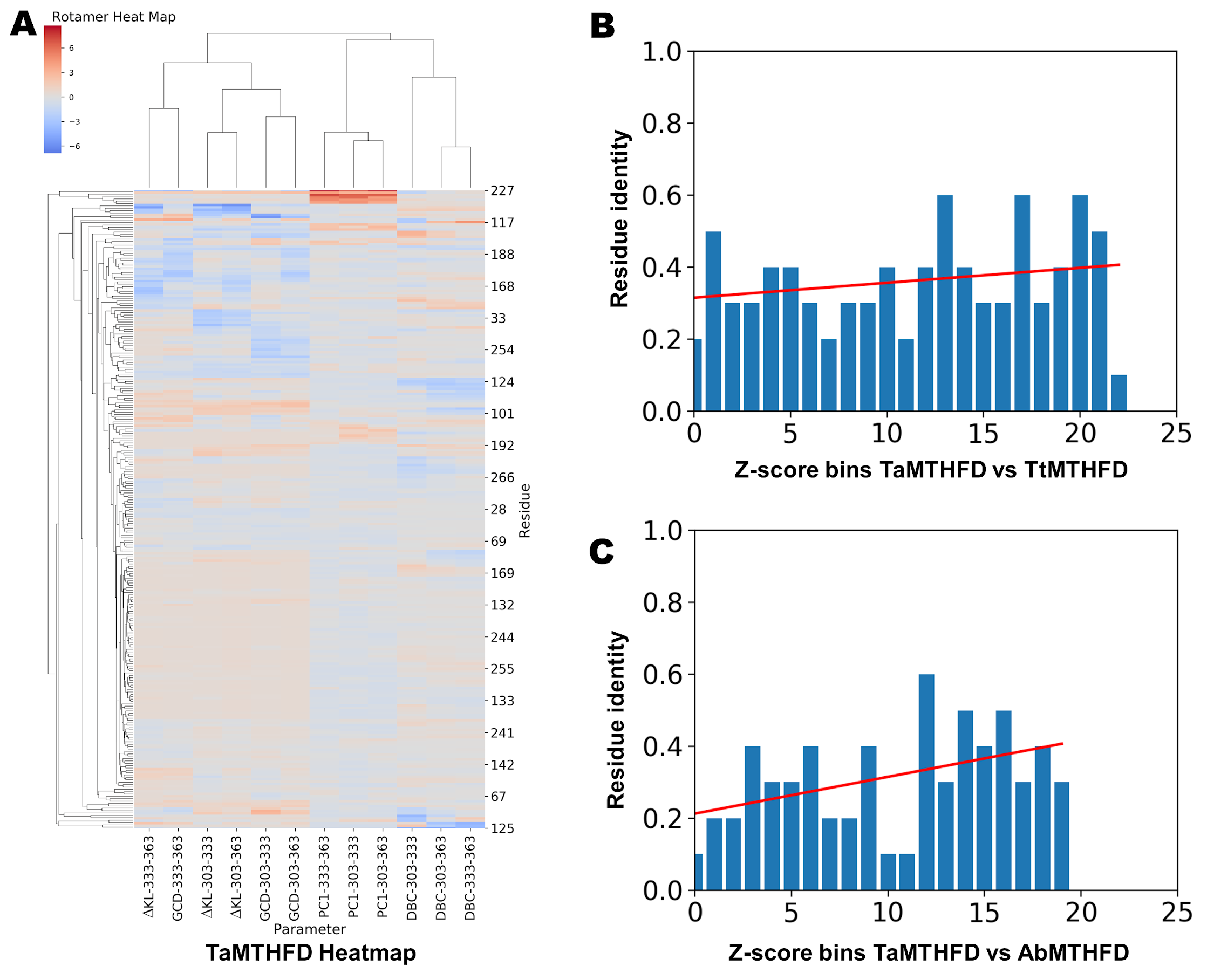

Supplement: S1 Fig — (A) PC1, DBC, GCD and ΔKL values were hierarchically clustered for the T. acidophilum MTHFD MD simulations at the indicated temperatures. Residue identity histograms comparing T. acidophilum MTHFD with T. thermophilus MTHFD (B) and with A. baumannii MTHFD (C). The less thermophilic TaMTHFD showed only a small trend to have more substituted amino acids at high composite Z-score positions when compared with TtMTHFD and had a more significant trend when compared with AbMTHFD. (TIF) [file pone.0232959.s001.tif]

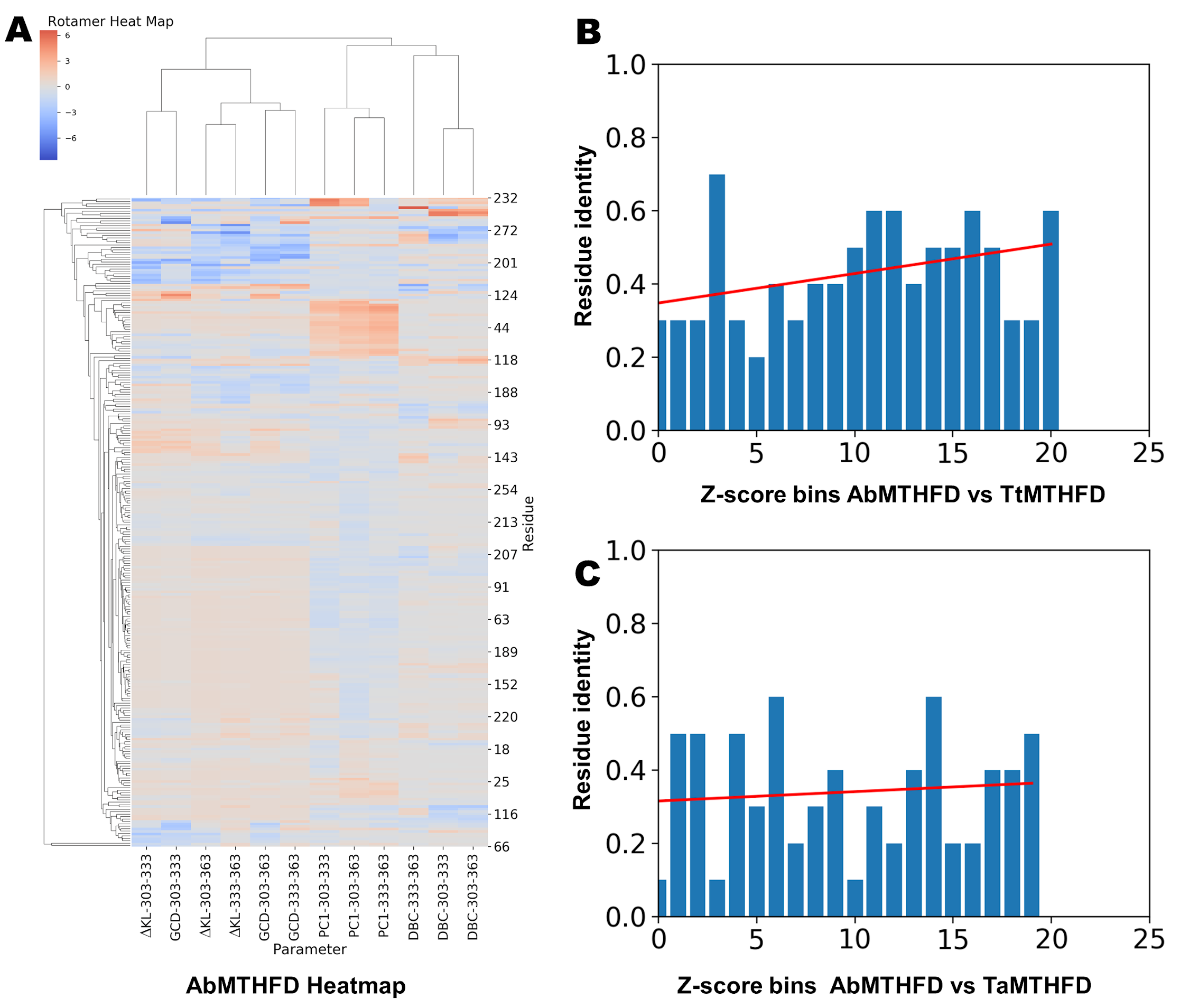

Supplement: S2 Fig — (A) PC1, DBC, GCD and ΔKL values were hierarchically clustered for the A. baumannii MTHFD MD simulations at the indicated temperatures. Residue identity histograms comparing A. baumannii MTHFD with T. thermophilus MTHFD (B) and with T. acidophilum MTHFD (C). AbMTHFD showed a trend to have more substituted amino acids at high composite Z-score positions when compared with TtMTHFD and had a slight trend when compared to TaMTHFD. (TIF) [file pone.0232959.s002.tif]
